# Effect of Disclosed Information on Product Liking, Emotional Profile, and Purchase Intent: A Case of Chocolate Brownies Containing Edible-Cricket Protein

**DOI:** 10.3390/foods10081769

**Published:** 2021-07-30

**Authors:** Cristhiam E. Gurdian, Damir D. Torrico, Bin Li, Georgianna Tuuri, Witoon Prinyawiwatkul

**Affiliations:** 1 Agricultural Center, School of Nutrition and Food Sciences, Louisiana State University, Baton Rouge, LA 70803, USA; cgurdi3@lsu.edu (C.E.G.); gtuuri@agcenter.lsu.edu (G.T.); 2 Department of Wine, Food and Molecular Biosciences, Faculty of Agriculture and Life Sciences, Lincoln University, Lincoln 7647, New Zealand; damir.torrico@lincoln.ac.nz; 3 Agricultural Center, Department of Experimental Statistics, Louisiana State University, Baton Rouge, LA 70803, USA; bli@lsu.edu

**Keywords:** sentiment analysis, alternative insect protein, anthropo-entomophagy, consumer behavior, extrinsic-product cue, cognitive information

## Abstract

Edible insects, a sustainable and nutritious alternative to conventionally derived proteins, are unfamiliar to Westerners and often associated with negative sentiments. Edible-cricket protein (ECP) added to chocolate brownies (CB) [0% ECP = CBWO (without) vs. 6% *w/w* ECP = CBW (with)], and disclosed information [no ECP added = (−) vs. ECP with benefits = (+), ECP− and ECP+, respectively] yielded four CB treatments (CBWO−, CBWO+, CBW−, and CBW+). Subjects (*n* = 112 female and *n* = 98 male) rated liking, selected emotions before- and after-tasting, and determined consumption (CI) and purchase intent (PI) after tasting. Likings were analyzed with mixed-effects ANOVA and post hoc Tukey’s HSD test. Emotions were evaluated with Cochran’s-Q test and correspondence analysis. Emotions driving or inhibiting overall liking (OL) were assessed with penalty-lift analyses using two-sample *t*-tests. A random forest algorithm was used to predict PI and estimate variables’ importance. Female’s and male’s expected OL were higher for CBWO− than for CBWO+. Females’ actual OL was higher for CBWO than for CBW regardless of the disclosed information but males’ actual OL was the same across treatments. Females exhibited negative-liking disconfirmation for CBW−. In both tasting conditions, the disclosed information affected treatments’ emotional profiles more than formulation. After-tasting emotions “happy” and “satisfied” were critical predictors of PI.

## 1. Introduction

The expected rise in the global population has increased the need for finding more efficient ways to obtain nutrient-dense sustainable foods [1,2]. Presently, protein deficiency is a leading cause of malnutrition for over one billion people worldwide [3]. Thus, investigations of ways to achieve a sustainable protein supply are being conducted, which includes using new technologies and ingredients to produce protein-rich foods [4]. For instance, edible insects can be produced with a higher feed-conversion efficiency, lower spending of environmental resources (e.g., water, land, feed), and less ecosystem pollution than conventional-source-derived proteins (including plant-based and livestock) [5]. Overall, edible insects have a high-quality nutritional profile, which depends on species, developmental stage, diet, sex, and processing among other variables [6,7,8]. Hence, functional ingredients could be obtained from their protein, fat, and chitin components. Particularly, the incorporation of edible insect protein in foods will be governed by the functionality they can add to the formulations; hence, there is a growing interest in studying their physio-chemical properties and sensory acceptability in different food categories. Although insects represent an eco-friendly and nutritious alternative to conventionally derived protein sources, the available information regarding their safety as a food ingredient is still limited [5]. According to Murefu et al. [9], the potential food safety hazards associated with edible insects are of chemical, biological, and allergenic nature (arginine kinase, α-amylase, tropomyosin, and other proteins also present in crustaceans which are widely known allergens in edible insects). The extent to which contaminants negatively affect the safety of edible insects as foods is determined primarily by the production system, species, developmental stage at harvest, and feeding (including sources) in the rearing process. This suggests that controlled conditions rather than wild harvesting shall be practiced to guarantee adequate food safety standards in edible insects [5].

Acceptable food products containing edible insect ingredients in bakery [8,10,11,12,13,14,15], energy/protein bars [16], extruded snacks [17], pasta [18], and meat [19,20] categories have been reported. However, there is still a significant reluctance to consume edible insect food products mainly in western cultures, where entomophagy is not a common practice [21]. Such rejection has been associated primarily with disgust [22] and neophobia [23]. These challenges could be addressed or at least counterbalanced by educating consumers about the benefits of edible insects [24], introducing novel food products with a “similarity of tasting” approach (i.e., tastes like another popular product) [19], incorporating edible insects or their nutrient fractions as “invisible ingredients,” such as flours, extracts or powders [25] in familiar products [26], and promoting tasting experiences with edible-insect products to improve consumers’ familiarity [27]. Different food matrices can be used to study the incorporation of novel ingredients in food products. For instance, chocolate brownies (CB) are familiar to consumers, highly acceptable, and commonly associated with positive feelings, which makes CB an appropriate food model for the incorporation of edible insect products [28,29,30]. Edible cricket protein (ECP) is a high-quality protein produced in more sustainable conditions than plant or animal-based proteins, but has not been sufficiently explored regarding its acceptability in the US marketplace [31]. Yet, Fischer and Steenbekkers [32] reported that Westerners are more receptive to crickets, mealworms, and grasshoppers than to other edible insects.

Predicting consumers’ food choice with models based solely on hedonic information may not yield adequate prediction power compared to more holistic models that incorporate product-elicited emotions information [33]. In sensory studies, the collected data are usually analyzed via multivariate projection techniques such as principal component analysis, to describe the treatments and/or explain the observed differences among them [34]. However, predictive discriminant models can be built on sensory and emotional data to efficiently discriminate among treatments and to provide a measure of variable importance for future sensory analysis applications [35]. Recently, machine learning and data mining have become more popular by providing modeling tools to predict variable outcomes based on ensembles of predictors, such as random forest (RF) and bootstrap-aggregation (bagging) trees, that perform better than their single predictors [36]. To the best of our knowledge, these tools have not been fully explored to model sensory and emotional data together with demographic information. The inclusion of emotions (before- and after-tasting) evoked by CB formulations without and with ECP upon disclosing ECP presence and its benefits to consumers in addition to product acceptance and other demographic and experimental variables may improve the performance of an RF model predicting purchase intent (PI) and aid marketing strategies for the introduction of edible-insect foods into the US marketplace.

The effect of product benefit claims, such as sustainability or high-nutritional value on the PI, emotions, and overall liking (OL) has been widely studied in different products. The effect of the claims varies depending on the food category, implied benefits, and the population being studied [34,37]. Several studies have reported the positive effects of disclosed benefit claims on consumer acceptability, perception, PI, or emotional profiles [38] albeit others have found them irrelevant [39] or not significant for certain demographic groups [40]. To our knowledge, the effect of disclosing the presence of ECP in CB while communicating the sustainability and nutritional-quality benefits derived from its consumption on product acceptability and emotional profiles as they relate to PI has not yet been studied. Therefore, the objective of this study was to investigate whether disclosing ECP presence accompanied by an environmental and nutritional-quality claim affected the expected (before-tasting) and actual (after-tasting) OL, emotional profiles, and/or PI of CB formulations (CBWO and CBW).

## 2. Materials and Methods

### 2.1. Chocolate Brownies (CB) Preparation

Chocolate brownies (CB) were prepared with Betty-Crocker fudge batter mix comprising sugar, enriched flour bleached (wheat flour, niacin, iron, thiamin mononitrate, riboflavin, folic acid), cocoa processed with alkali, palm oil, corn syrup, corn starch, and 2% or less of: carob powder, salt, canola oil, and artificial flavor (General Mills Sales, Inc., Minneapolis, MN, USA), USDA grade A large-white eggs (Great Value, Walmart Stores, Inc., Bentonville, AR, USA), and Wesson canola oil (Conagra Brands, Chicago, IL, USA). Edible cricket protein (ECP) commercialized as Griopro 100% cricket powder (All Things Bugs LLC, Midwest City, OK, USA) made of whole crickets (*Acheta domesticus and Gryllodes sigillatus*) containing 65% *w/w* protein, 22.5% *w/w* fat, and 5% total carbohydrate (wet basis) was added (6% *w/w*) to the formulation. This concentration of ECP was based on preliminary data from a trial with 25 subjects tasting CB within a range of ECP (3–10% *w/w*) for which 6% *w/w* was the highest percentage before significant taste and aroma rejection occurred due to an earthy off-flavor/aroma and/or a rancid aftertaste. Batches of each CB formulation (without ECP, CBWO, and with 6% *w/w* ECP, CBW) were prepared the day before the consumer study. Briefly, eggs (875 g), water (258 g), canola oil (621 g), batter mix (3128 g), and ECP powder (312 g, only added for CBW) were stirred together in a Globe SP20 commercial food mixer (Globe Food Equipment CO, Dayton, OH, USA) at speed 2 for each batch. The mixture was then placed in a 45.7 cm × 66 cm aluminum tray and baked in a pre-heated OV310G mini rotating rack oven (Baxter Mfg, a Division of ITW FEG, LLC, Orting, WA, USA) at 325 °F for 52 min. Baked CBWO and CBW were stored separately at room temperature in food grade BPA-free polypropylene 2 oz. clear plastic-lidded portion cups (CrystalWare, Lakewood, NJ, USA) overnight until the consumer study was performed.

### 2.2. Consumer Study

The research protocol was approved by Louisiana State University (LSU) Agricultural Center Institutional Review Board (IRB # HE 18-9 and # HE 18-22). Participants (*n* = 210 untrained consumers 18 years of age and older; Table 1) were recruited from a pool of faculty, staff, and students at the LSU campus, Baton Rouge, LA, USA. Recruitment criteria included: (1) no self-reported allergy or adverse reactions towards any ingredients of the CB samples or unsalted crackers, (2) willingness to taste samples that contain edible cricket protein (ECP) powder, (3) absence of any physiological or medical conditions that would compromise their performance in the sensory evaluation, and (4) self-reported regular consumption (at least once per month) of CB. Subsequently, subjects agreed with and signed a consent form included in the approved research protocol.

### 2.3. Questionnaire: Consumer Liking, Emotions, Consumption (CI) and Purchase Intent (PI)

**Figure 1:**
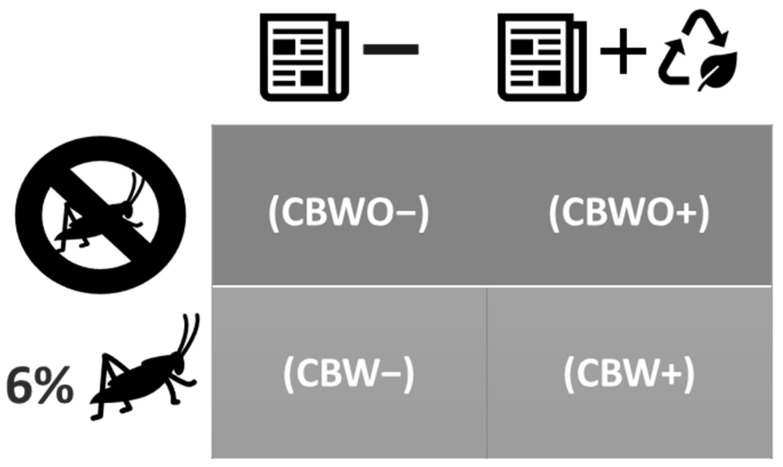
Factorial arrangement for the chocolate brownie (CB) treatments. ECP = Edible-cricket protein. WO: Without; W: With. ECP−: “No ECP added” disclosed information; ECP+: “Contains ECP + benefits” disclosed information. (1) CBWO− = CB without ECP (CBWO) presented under the ECP− disclosed information; (2) CBWO+ = CBWO presented under the ECP+ disclosed information; (3) CBW− = CB with 6% ECP (CBW) presented under the ECP− disclosed information; (4) CBW+ = CBW presented under the ECP+ disclosed information.

Qualtrics software (Qualtrics, Provo, UT, USA) was used to administer the computer-based questionnaires given to panelists and to collect their responses. The four CB treatments (Figure 1) were presented together before starting the evaluation. Then, consumers were instructed to evaluate them in a monadic-sequential order as indicated on the screen based on the three-digit sample blinding code, and specific sample information and related benefits of ECP were given for each sample. The full related benefits of ECP statement was as followed “This sample contains ECP. Edible insects are safe to eat and are considered a sustainable source of high-quality protein and other nutrients. Edible insect production has less negative environmental impact than traditional livestock production. An estimated two billion people worldwide consume edible insects” [26].

The experimental design was a completely randomized block design (CRBD) with a factorial arrangement. The formulation and disclosed information factors had 2 levels each (formulation levels: without ECP and with 6% ECP; disclosed information levels: “No ECP added” statement and “Sample contains ECP and benefits” statement). The Qualtrics software instructed the panelists which of the treatments shall be evaluated first and disclosing absence of ECP or alternatively its presence accompanied by its benefits was part of the treatment identity. The presentation order for the four treatments was balanced and randomized, so each of them had the same chance of being present in all four possible positions (half of the consumers evaluated first those treatments that contained the “this sample has ECP and benefits” information whereas the other half evaluated first those treatments that contained the “No ECP was added” information. Specifically, when consumers evaluated CBWO− (treatment 1) first, it involved a CB without ECP accompanied by a “No ECP was added to this sample” statement; when they evaluated CBWO+ (treatment 2) first, it involved a CB without ECP accompanied by a “This sample contains ECP and the nutritional and environmental benefits from ECP”; when they evaluated CBW− (treatment 3) first, it involved a CB with 6% ECP accompanied by a “No ECP was added to this sample”; finally, when they evaluated CBW+ (treatment 4) first, it involved a CB with 6% ECP accompanied by a “This sample contains ECP and the nutritional and environmental benefits from ECP”. The treatments were evaluated in two experimental conditions (before- and after-tasting) in one sensory session.

The evaluation consisted of (1) reporting elicited emotions before tasting (based on the sample’s visual evaluation and the disclosed information) on a Check-all-that-apply (CATA) basis from a list of twenty-five emotion terms from the Essense25 profile emotion word list [27]; (2) rating expected (before-tasting) likings with a 9-point-hedonic scale (left-anchored dislike extremely and right-anchored like extremely); (3) reporting elicited emotions upon tasting on the CATA list mentioned above; (4) rating actual (after-tasting) likings with the previously mentioned 9-point-hedonic scale; and (5) indicating consumption intent (CI) and purchase intent (PI) if the sample were commercially available with a binomial scale (Yes or No).

### 2.4. Statistical Analysis

The sensory evaluation of CB treatments (resulting from the 2 × 2 factorial arrangement of formulation and disclosed information levels) followed a balanced and randomized block design (panelists as blocks). Statistical data analysis was conducted using the Statistical Analysis Software (SAS) version 9.4 (Cary, NC, USA), R software version 4.0.3 (RStudio, Inc., Boston, MA, USA), and the XLSTAT (Addinsoft, New York, NY, USA) statistical software version 2020 [41] with α = 0.05 significance level. The effect of formulation (CBWO vs. CBW), disclosed information (ECP− vs. ECP+), demographics, tasting condition (before vs. after) and up to three-way interactions between gender (females vs. males), formulation, and disclosed information and between tasting condition, formulation, and disclosed information on overall liking (OL) was investigated with multi-way analysis of variance (ANOVA) in a mixed-effects model having panelists as a random effect and Tukey’s HSD post hoc test. Check-all-that-apply (CATA) binary data from emotions (before- and after-tasting) were analyzed according to the procedures reported by Meyners et al. [42] and Ares et al. [43] segmented by tasting condition and gender. Global/individual Cochran Q tests determined the overall/individual effect of treatments within tasting condition and tasting condition within treatment in emotions distribution/each emotion term frequency distribution. Subsequently, all pairwise comparisons were conducted for treatment groups as well as tasting conditions following the Marascuilo and McSweeney procedure based on minimum required difference [44]. The proportion of discriminant emotions across genders within tasting conditions and across tasting conditions within genders were compared with two-population proportions Z-tests and two-tailed McNemar tests for correlated proportions, respectively. Emotions (segmented by tasting condition and gender), consumption intent (CI) and purchase intent (PI) were then input to a correspondence analysis based on Chi-square distances. For each tasting condition (before and after) and gender, the relationship between elicited emotions and product liking was unfolded through penalty-lift analysis of before-tasting and after-tasting OL to identify drivers/inhibitors of product liking. Overall liking mean impact was calculated as the mean OL difference from present vs. absent categories for each emotion with a 20% population threshold [38]. This difference was then standardized, and its significance (*p* < 0.05) was tested with a two-sample *t*-test. The random forest (RF) algorithm, an ensemble of decision trees, which are combined to predict a single outcome and modelled to provide diversity between the trees [45] was used to model PI prediction (using mtry = 32 features out of 68 in the random selection at each node of the *n* = 1000 decision trees) from formulation, disclosed information, demographic variables, sensory likings (before- and after-tasting), emotions (before- and after-tasting), and CI using full data as interest was on model performance. Because RF is an ensemble of several low-bias-high-variance components (decision trees), the RF variance is reduced, and its resulting discrimination is on average more accurate than its individual components. To increase the diversity among the decision trees, RF fits each tree on a random subset of the dataset, of the same length, selected with replacement (bootstrap replicate). In addition, diversity is increased during the growing of the trees as for each node, RF picks a small random subset of predictor attributes and uses only this subset to search for the best split of the data into their observed classes or numerical outcome. A noteworthy feature of RF is the overfitting control. Although RF can be composed of a large number of decision trees, the error rate for new samples converges to a limiting value when the number of trees goes to infinity [35]. The misclassification rate for RF was estimated using the out-of-bag observations and the classifier’s performance was displayed on the Receiver Operating Characteristic (ROC) curve. Plots of variables relative importance from RF were obtained based on the mean decrease in accuracy and mean decrease in Gini index, which measures node impurity for classification trees.

## 3. Results and Discussion

### 3.1. Significance of Main Effects in Product Liking

The significance of the main effects and their interactions of interest (up to 3-way) on treatments’ OL is summarized in the analysis of variance (ANOVA) shown in Table 2.

Tasting condition, formulation, and their 2-way interaction were significant (*p* < 0.05) for OL. Disregarding all other effects, OL was significantly (*p* < 0.05) lower after-tasting (6.30) than before-tasting (6.55) and was significantly (*p* < 0.05) lower for CBW than for CBWO (6.26 vs. 6.60, respectively). The levels of formulation (CBWO vs. CBW) influenced the way subjects rated their OL for treatments depending on the tasting condition (before vs. after tasting). Although the OL ratings were not significantly (*p* = 0.08) influenced by the levels of disclosed information (ECP− vs. ECP+), there was a significant (*p* < 0.05) interaction of disclosed information with tasting condition. On the other hand, gender levels (female vs. male) significantly (*p* < 0.05) interacted with the formulation effect causing differences in the OL ratings. Previous research indicated that males exhibited higher acceptability for edible insects than females [31,46] possibly because they had lower disgust sensitivity, experienced more curiosity, or associated novelty with edible insects more than females, which drove their willingness to try and ultimate acceptability of edible insects.

### 3.2. Effects of Formulation, Disclosed Information, and Gender on Expected and Actual Overall Liking

**Figure 2:**
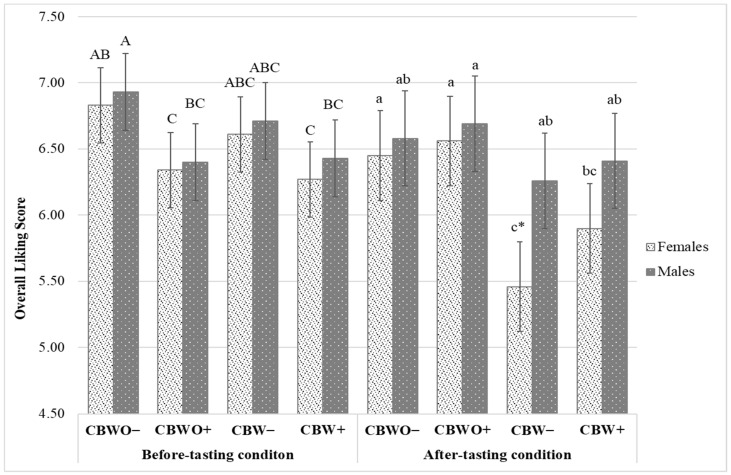
Treatments overall liking (OL) bar chart segmented by tasting condition (before vs. after tasting). Data are OL least square means and standard errors from *n* = 112 female and *n* = 98 male groups. Treatments are described in Figure 1. Different uppercase/lowercase letters indicate significantly (*p* < 0.05) different before tasting/after tasting OL scores (Tukey’s means separation) across treatments and gender. * Denotes significantly (*p* < 0.05) lower after-tasting OL score than its corresponding before-tasting OL score (Tukey’s means separation).

Figure 2 shows the treatments’ OL least-square means in the before (expected) and after (actual) tasting conditions from the female and male groups. The CBWO expected OL was negatively affected (*p* < 0.05) by the ECP+ disclosed information in both genders, which could be attributed to food neophobia [47], disgust feeling [48], and other product-elicited mental associations with unpleasant variables [22]. Food neophobia is mainly related to unfamiliarity with novel foods while disgust is thought to be originated from mental associations with other disgusting variables, which makes it more complex to be understood and overcome or counterbalance. Both negative-product-elicited traits are considered the major limitation for the willingness to try edible insects in Western societies [21,49,50] although La Barbera et al. [22] found them uncorrelated and determined that “disgusting” feelings were more important than neophobia when predicting the willingness to eat insects. Although ECP+ disclosed information communicated environmental and nutritional benefits associated with anthropo-entomophagy, the negative feelings and expectations exerted a stronger effect than the environmental or nutritional consciousness and positive sensations. Possibly, sustainability and nutritional consciousness were not significant drivers for the expected OL of CB containing ECP [49]. On the other hand, the formulation had no significant effect (*p* > 0.05) on OL expectation regardless of the disclosed information. The perceived difference in appearance among formulations was not large enough to yield significant differences in liking expectations.

In the after-tasting condition, the female group rated a significantly higher (*p* < 0.05) OL for CBWO than for CBW for either disclosed information, but the male group rated similar (*p* > 0.05) OL across formulations for either disclosed information. The female group’s mean OL (5.46) was significantly (*p* < 0.05) lower than that of the male group (6.26) only for CBW−. Possibly, the female group presented a lower taste rejection threshold than the male group for ECP, which suggests males are more likely to accept products containing ECP than females. Previous studies have found similar results claiming males had a lower aversion to consuming products containing edible insects than females [46,50,51,52,53]. However, other studies have suggested food neophobia [54], disgust [55], indirect (via disgust effect) implicit attitudes derived from implicit associations with edible insects [22], social and cultural norms [31], and perceived behavioral control [56] rather than gender as stronger determinants for the willingness to consume insects and actual-consumption behavior. Lower perceived behavioral control, higher measurements for neophobia and disgust, and more traditional food culture decrease the likelihood of edible insect consumption.

The disclosed information had no significant effect (*p* > 0.05) on actual OL ratings for either group (female and male) and either formulation (CBWO and CBW).

Other authors have also concluded that communicating environmental and health benefits of entomophagy is insufficient to alter the sensory acceptability of foods containing edible insects [25,46,57]. When consumers evaluate (taste/interact) products, their expectations for a given attribute or product’s performance can be met (if actual performance after interacting with the product is as expected) or disconfirmed (negatively when intensity/liking expectations are higher than the actual perceptions/likings, or positively when they are higher than the intensity/liking expectations). When disconfirmation occurs, product acceptability can be: (1) aligned with expectations, (2) affected (positively or negatively) to a greater extent than if expectations had not been present, (3) negatively affected regardless of the direction of the disconfirmation, or (4) assimilated/contrasted with expectations depending on the perceived magnitude of the discrepancy [58]. Moreover, when sensory expectations are negatively disconfirmed, the probability of repeated purchase/consumption may decrease [57]. Comparing the before and after-tasting scenarios, CBW− had a significant (*p* < 0.05) negative liking disconfirmation among the female group, but the OL expectations for CBW+ were not significantly (*p* > 0.05) disconfirmed upon tasting (Figure 2). This suggests a positive effect of the disclosed information [24,59], which is possibly associated with the subjects’ degree of environmental or nutritional consciousness [51]. The significant negative disconfirmation observed in the female group for CBW− could be explained by the deception caused by ECP− (appearance of CBW and claim of ECP absence made them believe they would taste and experience the characteristic sweet and chocolate flavors from regular brownies but instead they tasted ECP− added flavor). In the female group, the experienced discrepancies between the CBW− expected OL and the OL perceived after tasting were sufficiently large leading CBW− into a rejection region. In this region, an increase in the perceived real difference resulted in an under-rated actual OL when compared to a scenario without expectations. On the contrary, ECP+ disclosed information “prepared” the female group to experience the sensory characteristics of ECP (based on experience, beliefs, or mental associations) so no negative disconfirmation occurred for CBWO+ or CBW+. Rather, an assimilation effect was observed for CBWO+, in which the perceived OL after tasting was matched to the expected OL.

Overall, actual OL scores of at least 7 on a 9-point-hedonic scale are considered promising for regular food products [60] but given ECP represents a new concept for Westerners, the obtained actual OL for CBW+ (female group = 5.90 and male group = 6.41) represents an encouraging starting point for the incorporation of ECP into similar bakery products especially if targeting male consumers [28]. Moreover, the way information is conveyed can affect the consumers’ perceptions and liking. In this study, the ECP−associated benefits were presented in the form of a statement accompanied by a picture of the ECP, but delivering the same information on the packaging or in informative sessions could improve the actual acceptability of CBW+ [56].

### 3.3. Effects of Formulation and Disclosed Information on Emotional Profiles before and after Tasting

#### 3.3.1. Emotional Profiles of Males vs. Females before Tasting

The treatments’ emotional profile based on self-reported applicable emotion terms from the Essense25 list [61] was evaluated separately for each gender and tasting condition. In the before-tasting condition, the female group exhibited a significantly (*p* < 0.05) higher proportion (17/25) of discriminant emotions than the male group (6/25). Other researchers have also reported higher emotional discrimination for food products among females when compared to males [62]. Table 3 shows the emotional profiles from the before-tasting condition exploring the observed differences between treatments separately for each gender.

For the female group, the ECP+ disclosed information led to a significant (*p* < 0.05) increase in the frequency of “adventurous,” “interested,” and “wild” regardless of the formulation while reducing the observed frequency of “bored” only for the CBW formulation. Similarly, the ECP+ disclosed information increased the frequency of the “adventurous” and “wild” emotions for both formulations among the male group and reduced the frequency of “bored” only for CBW. This pattern of emotional terms is common for individuals seeking pronounced sensations [63]. Sensation seeking is considered a powerful predictor of edible insect acceptability [49], exhibiting a strong positive correlation (0.30) with the acceptability of insect flour in foods [59]. Interest in the environment together with neophobia, familiarity, convenience, and affinity for meat are considered determinant variables for the readiness to adopt edible insects [64]. Neophobic subjects unconcerned with the environmental impact of food choices and with a high affinity for meat-based diets are less likely to adopt edible insects [51]. Still, presenting edible insects as invisible ingredients in familiar food products [19] with an appropriate sensory profile has been effective to improve their willingness to try [65].

Nevertheless, disclosing the presence of ECP and its benefits (ECP+) in CB also elicited unfavorable effects for both genders before-tasting emotional profiles. For the female group, a significant (*p* < 0.05) decrease in the proportion of “good,” “happy,” and “safe” positive emotion terms occurred for CBW+ when compared to CBW− while “worried” occurred more frequently for ECP+ disclosed information than for ECP− for either formulation. Similarly, for the male group, ECP+ significantly (*p* < 0.05) decreased the “calm” and “safe” terms and increased the choice frequency for “worried” for CBW. Additionally, ECP+ decreased the frequency of the “warm” term for the male group for CBWO when compared to ECP−. The observed negative effect of ECP+ triggering unsafety, mental discomfort, and lack of confidence in both genders agrees with other studies reporting “worry” and “concern” emotions from individuals regarding their safety (health risks) when eating foods containing edible insects [5]. These concerns arise mainly because of the limited availability of information about the process used to guarantee the innocuity and quality of the insect-derived ingredient [48,66] and its regulations [67] when incorporated into foods. However, this could be substantially improved if potential consumers are educated about the safety and regulations governing edible insect process throughout the added-value chain starting in farms until presented in a meal [68] and by repeated exposure to tasting events involving edible insects without any health-related adverse outcome [69].

**Figure 3:**
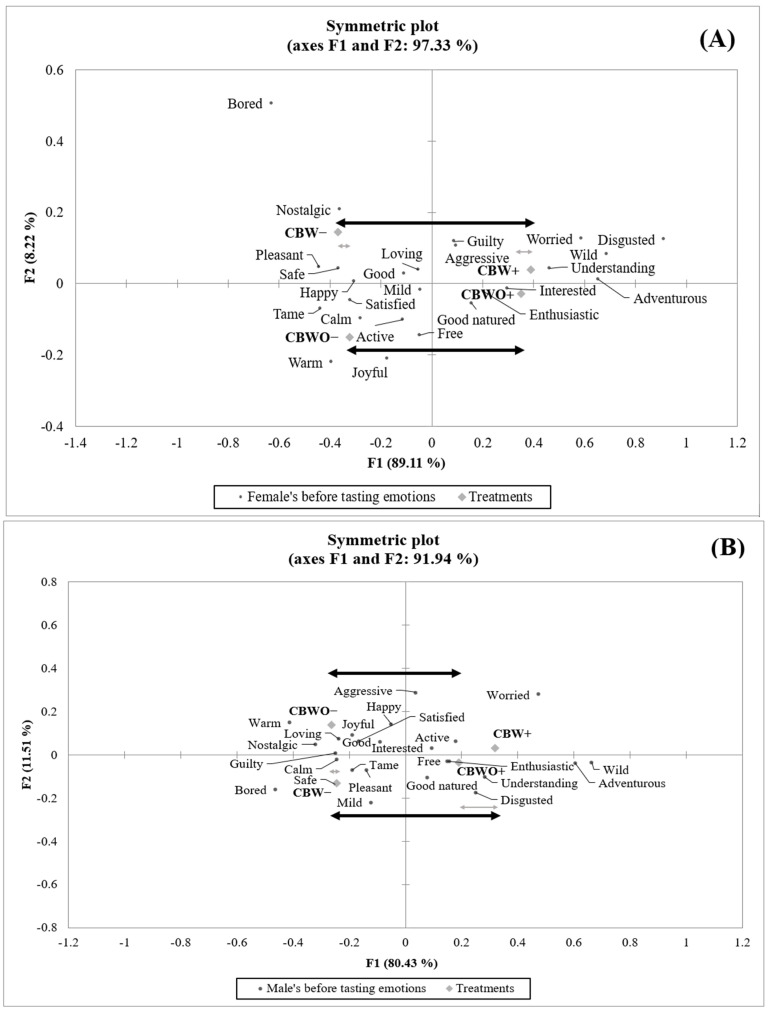
Correspondence analysis (chi-squared distance) symmetric plot visualizing treatments and emotions in the before-tasting condition from (**A**) female (*n* = 112) and (**B**) male (*n* = 98) groups. Treatments are described in Figure 1.

In the female group, the “calm” and “tame” emotions were selected fewer times when CBWO was presented under ECP+ disclosed information; yet this effect is difficult to interpret as it could be both, positive and negative because it could reflect an “energetic” but also “nervous” or “anxious” short-term response or long-lasting state [70]. In fact, other researchers have categorized the “tame” emotion as an unclassified term [71,72]. Another adverse effect of the ECP+ disclosed information among the female group was the decreased frequency of the “pleasant” emotion’s proportion for both formulations and increased frequency of the “disgust” term for CBWO when contrasting against ECP−. Disgust sensitivity has been identified as one of the major and most challenging constraints to entomophagy in the Western world [49], which is more frequent in young [73] females than in male consumers [74]. Overcoming disgust is key to improve the willingness to eat and/or buy insect foods because it is one of its most important predictors [75]. On the other hand, treatments’ emotional profile in the before-tasting condition showed a minimal effect of formulation for either gender (Figure 3A,B).

#### 3.3.2. Emotional Profiles of Males vs. Females after Tasting

Contrary to the before-tasting condition, the proportion of discriminant after-tasting emotion terms for the female group (9/25) was not significantly different (*p* > 0.05) from that of the male group (4/25). Table 4 shows the effect of formulation and disclosed information on the treatments’ emotional profile in the after-tasting condition by gender. For the female group, the “adventurous” and “interested” emotions were positively affected by the ECP+ disclosed information in both formulations, and the “bored” emotion was less frequent for CBWO+ than for CBWO−.

On the other hand, the male group was positively influenced by the ECP+ disclosed information for both formulations regarding the “adventurous” and “wild” emotions, which belong to the *active* dimension (which reflects characteristic emotions of an “energetic” state or mood elicited upon tasting foods and/or reading food names) [76] while the “bored” term was less frequent for CBW+ than for CBW−, which is generally considered a negative term with a high arousal state that commonly decreases food liking and intake [77]. These results suggest that an appropriate marketing campaign for ECP should lie in the context with novelty, adventure, and wild sensations [49,78].

The “understanding” emotion in the female group became more frequent for CBW when presented with the ECP+ disclosed information than when presented with the ECP− disclosed information. Although “understanding” emotion has been considered an unclassified term in some studies [72], others have placed it in the positive dimension or have found a significant positive correlation between “understanding” and product liking [79,80,81]. In this study, the female group possibly felt more understanding about the sensory profile of CBW+ (different flavor notes and texture characteristics compared to a regular brownie) because they were informed that ECP was present in the formulation. CBW− exhibited a lower proportion of the “understanding” emotion among the female group because of the disconfirmed sensory profile experienced for this treatment, which agrees with the observed behavior in the OL ratings previously discussed.

**Figure 4:**
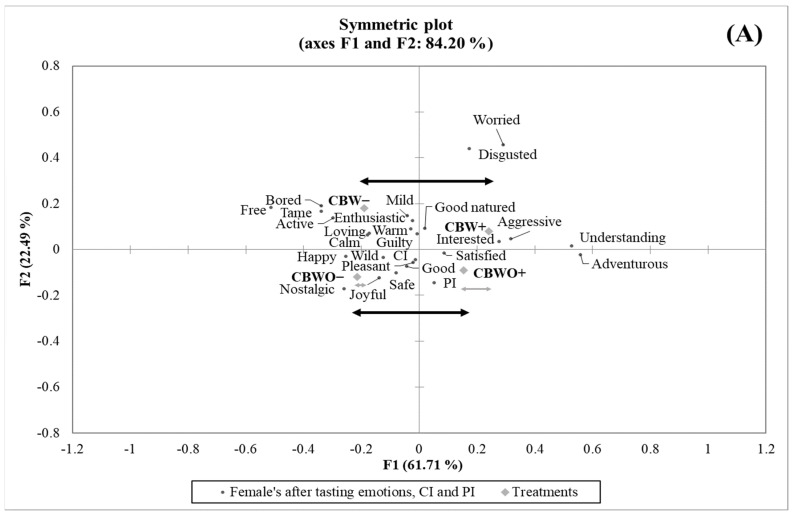

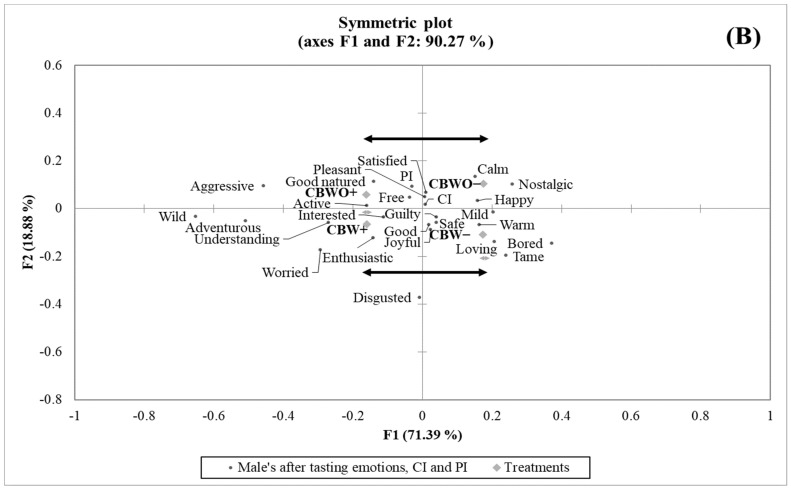
Correspondence analysis (chi-squared distance) symmetric plot visualizing emotions in the after-tasting condition, consumption intent (CI), purchase intent (PI), and treatments from (**A**) female (*n* = 112) and (**B**) male (*n* = 98) groups. Treatments are described in Figure 1.

However, the female group’s “free” emotion was negatively affected by the ECP+ disclosed information in the CBW formulation while the “calm” term significantly (*p* < 0.05) decreased in CBWO when presented under the ECP+ disclosed information compared to when presented under ECP−. Although the “worried” emotion was most frequent for CBW+ among the female group, it was not significantly (*p* > 0.05) different from CBWO+ or CBW−, evidencing an effect of the interaction between formulation and disclosed information. A formulation effect was observed among the female group only for the “good” emotion, which was significantly (*p* < 0.05) less frequent for CBW+ than for CBWO+. Still, among both groups (female and male), the disclosed information affected the treatments’ emotional profile in the after-tasting condition more than the formulation (Figure 4A,B).

#### 3.3.3. Differences in Emotional Profiles by Gender between Tasting Conditions

The female group exhibited significantly a higher (*p* < 0.05) proportion of discriminant emotion terms in the before tasting (17/25) condition than in the after tasting (9/25) condition, respectively, whereas the male group presented no significant (*p* > 0.05) differences in the proportion of discriminant emotions between tasting conditions (6/25 vs. 4/25 before- and after-tasting, respectively). This was expected as other researchers have reported a greater effect of informative claims on before-tasting elicited emotions [71].

Among the female group, the “adventurous” emotion significantly decreased upon tasting for both formulations (CBWO and CBW) when appearing with the ECP+ disclosed information, but for the male group, it decreased upon tasting only for CBW+. This could partially be explained by the need for optimization in CBW formulation; yet, since the effect was not observed for CBW−, it can also reflect bias triggered by the disclosed information or the need for a different/additional context of ECP+ emphasizing adventure, novelty, activeness, or a different product application closely related to “adventurous” feeling (e.g., energy drink, high-protein shakes, energy bars) [28,82].

Unexpected effects across tasting conditions were observed for both genders. The female group’s “bored” emotion significantly increased for both disclosed information (ECP− and ECP+) upon tasting but only for CBWO formulation, suggesting a positive effect of the CBW formulation. The female group selected “disgust” emotion more frequently in the after-tasting condition than in the before-tasting condition for CBWO− and CBW− whereas the male group presented a similar proportion of “disgust” emotion for all treatments across the before- and after-tasting conditions. Generally, females are likely to experience the “disgust” emotion more than males due to a higher disgust sensitivity [74]. On the other hand, the female group exhibited a decrease in the “enthusiastic” emotion upon tasting for all treatments and decreased “free” frequency for CBW+. The “good” emotion occurred more frequently in the after-tasting condition than in the before-tasting condition for CBWO+ among the female group and for CBW+ among the male group. Other studies have reported a lower likelihood for acceptability and/or willingness to consume edible insects for females [46,52,83] than for males.

Both genders exhibited an overall negative response towards all treatments upon tasting, which was evidenced by a decreased frequency of the “interested” emotion after-tasting when compared to the before-tasting condition. This behavior was possibly driven by a generalized negative state upon tasting disconfirmation regarding flavor, texture, or aroma characteristics that may or may not have affected the treatments’ likings but decreased their “interest” feeling. Alternatively, their curiosity regarding the sensory profile of samples or their identity was satisfied/deciphered upon tasting and their initial interest (before tasting) was mostly related to verifying their expectations. The “wild” and “worried” terms significantly decreased upon tasting only for the female group when presenting either formulation under ECP+ disclosed information. Schouteten et al. [84] reported that consumers elicited fewer negative emotions upon tasting insect-based burgers (insect ingredient was disclosed) when compared to the expected condition (ingredient was disclosed but no tasting took place), which supports our findings for the female group emotions of “worried” and “wild” towards CBWO+ and CBW+ upon tasting.

An overall positive effect of ECP+ for both genders was observed regarding “joyful” and “pleasant” positive-strong-valence emotions. The male group had an increased occurrence of the “joyful” and “mild” emotions upon tasting for CBW+ and CBWO−, respectively whereas in the female group, “pleasant” and “safe” emotions significantly decreased upon tasting for CBW− but, for CBWO+ and CBW+, the “pleasant” emotion increased significantly after tasting. The male group had a higher frequency of “pleasant” after tasting than before tasting only for CBWO+. Moreover, all treatments presented an increased frequency of “satisfied” emotion upon tasting for both genders (except for CBW− for the female group). King et al. [83] reported that males’ acceptability for food products was associated with “satisfied” and “disgust” emotions whereas for females “joyful,” “good,” “happy,” “pleasant,” and “disgusted” were accentuated out of the 25 emotions associated with acceptability.

### 3.4. Relationship between Product-Evoked Emotions and Liking

#### 3.4.1. Effect of Before-Tasting Emotional Profiles on Expected OL by Gender

**Figure 5:**
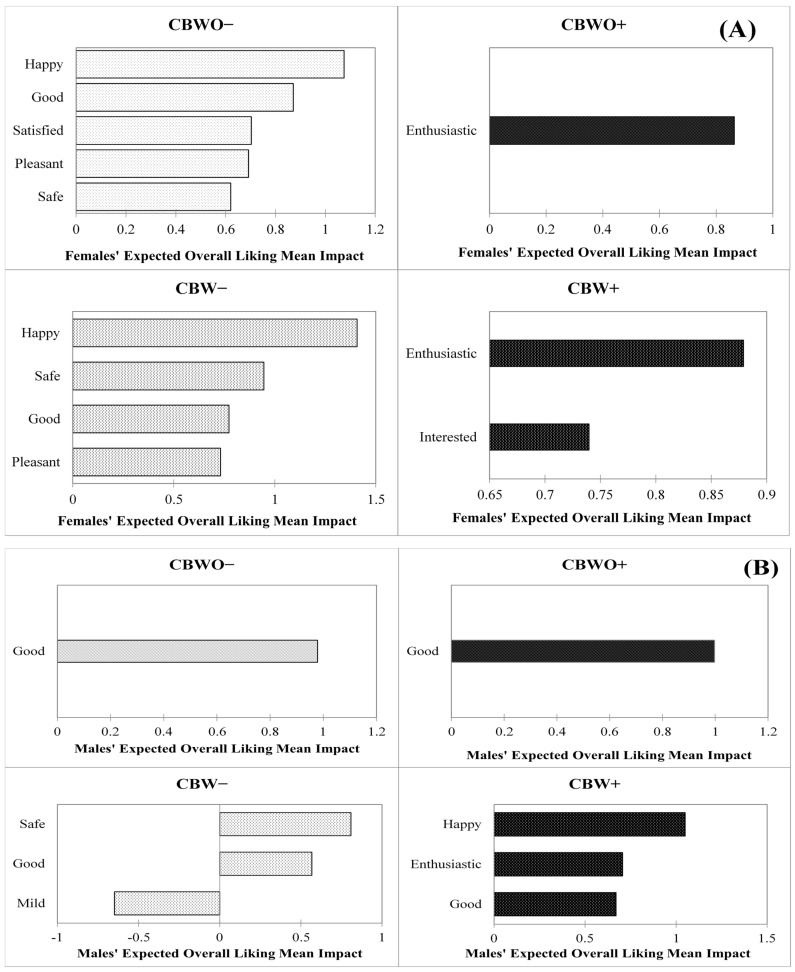
Treatments before-tasting overall liking (OL) mean impact (mean OL difference from present vs. absent categories for each emotion with a 20% population threshold size) vs. significant (*p* < 0.05, 2-sample *t*-test) emotions in the before-tasting condition (%) for (**A**) female (*n* = 112) and (**B**) male (*n* = 98) groups. Treatments are described in Figure 1.

Elicited emotions from the female and male groups in the before-tasting condition responsible for a significant (*p* < 0.05) effect on the expected OL of treatments are shown in Figure 5A,B, respectively. In the female group, the expected OL presented fewer and different significant emotion terms for either formulation when presented under the ECP+ disclosed information. Although ECP+ triggered a variety of emotions in both formulations, only a few of them significantly affected the expected OL [80]. Different formulations presented under the same disclosed information presented almost the same significant emotion terms. The emotions “happy,” “good,” “satisfied,” “pleasant,” and “safe,” for CBWO−, and “happy,” “safe,” “good,” and “pleasant” for CBW− positively affected the expected OL. Critical emotions for CBWO− and CBW− lie in the positive valence (pleasantness) dimension, which is strongly associated with product liking [80,84,85] and choice when involved with tasting [33] albeit “safe” is considered both, a positive and low activation/arousal emotion.

On the other hand, when both formulations were presented under the ECP+ condition, the “enthusiastic” (for CBWO+), and the “enthusiastic” and “interested” (for CBW+) emotions positively affected the expected OL. These feelings belong to the “sensation seeking” [86] emotions lying on the high activation/arousal dimension. High activation/arousal emotions together with liking, and valence emotions have strong predictive power for product choice based on extrinsic cues [33], but on their own, they are associated with the motivation state of wanting rather than liking [85]. For example, when feeling hungry, subjects tend to experience arousal emotions that assist in the food search. Contrariwise, low levels of emotional arousal are closely related to less food consumption [82].

This suggests that the female group may have perceived differences in the appearance between formulations, which made the term “safe” a more critical attribute for the expected OL of CBW− than for CBWO− and “interested” for CBW+ than for CBWO+.

Among the male group, emotions affecting the expected OL differed across the disclosed information only for the CBW treatments. The “good,” (positive-valence emotion) “safe,” and “mild” (low activation/arousal emotions) significantly affected CBW− expected OL [33]. The “happy” emotion (positively associated with the pleasantness dimension) enhanced CBW+ expected OL the most followed by the “enthusiastic” (high activation/arousal emotion) and the “good” (positive emotion) terms. Differences in critical emotion terms across formulations presented under the same disclosed information were due to the extra emotion terms present for CBW− (“safe” and “mild”) and CBW+ (“happy” and “enthusiastic”). The “good” positive emotion term positively affected the expected OL the most for CBWO for either disclosed information but was also critical for CBW− and CBW+.

#### 3.4.2. Effect of After-Tasting Emotional Profiles on Actual OL by Gender

**Figure 6:**
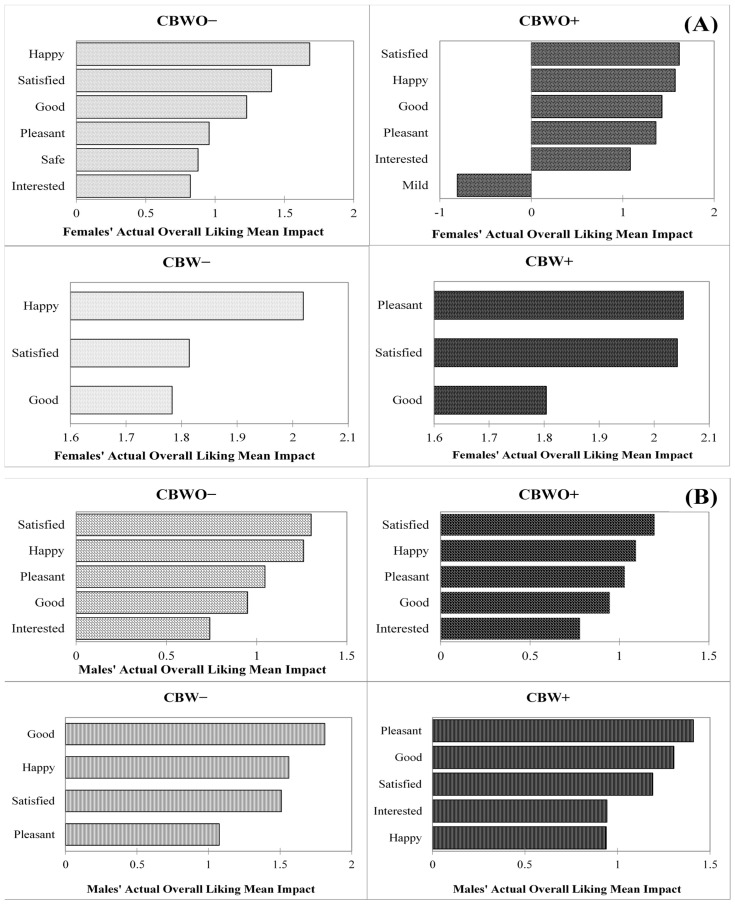
Treatments after-tasting overall liking (OL) mean impact (mean OL difference from present vs. absent categories for each emotion with a 20% population threshold size) vs. significant (*p* < 0.05, 2-sample *t*-test) emotions in the after-tasting condition (%) for (**A**) female (*n* = 112) and (**B**) male (*n* = 98) groups. Treatments are described in Figure 1.

Figure 6A,B illustrate elicited emotions in the after-tasting condition from the female and male groups that significantly (*p* < 0.05) affected treatments’ actual OL, respectively. Among the female group, the actual OL presented similar significant emotion terms across formulations for either disclosed information but for CBW treatments, fewer critical emotion terms affected the actual OL. When comparing across disclosed information, the “safe” and “mild” low activation/arousal emotions positively and negatively affected CBWO− and CBWO+ actual OL, respectively, while “happy” and “pleasant” positively affected CBW− and CBW+ actual OL for the female group, respectively. CBWO+ “mild” sensation reduced its actual OL possibly because female participants expected extravagant flavors or aroma from ECP, which were disconfirmed upon tasting. However, the disconfirmation experienced for CBW− did not elicit emotions that significantly inhibited its actual OL (considering a 20% selection threshold to evaluate significance). Although CBW− and CBW+ presented the lowest actual OL (5.46 and 5.90, respectively) within the female group (Figure 2) none of the after-tasting elicited emotions were significant inhibitors for it; on the contrary, the significant drivers for CBW− and CBW+ actual OL were all positive emotions in the valence continuum [87]. Product liking sometimes does not correlate well with emotions; products exhibiting low OL may elicit positive emotions and vice versa [80,83]. Nevertheless, liking and emotions together can better explain consumption behavior and food choices [76,88].

Among the male group, treatments presented the same drivers for actual OL except for CBW− (“interested” was not a significant OL driver), which belong to the positive valence dimension representing pleasantness and to the high activation/arousal dimension in the case of the “interested” emotion. The actual OL drivers for CBWO− and CBWO+ had the same order of importance whereas the order differed for CBW− and CBW+. These results further support the observed similarity in the male group’s actual OL (Figure 2) across treatments given that they shared similar critical drivers for the actual OL. Gutjar et al. [80] stated that emotions are weakly correlated with product acceptability because they provide further information not explained by liking. Hence, positive-valence emotions associated with pleasantness are common drivers of liking whereas low or high activation/arousal emotions are not associated with OL. This represents an interesting orthogonal dimension to liking that should be further explored to better understand consumers’ perceptions and behaviors [81].

### 3.5. Purchase Intent (PI) Predictive Importance of Socio-Demographic and Experimental Variables, Product-Evoked Emotions and Liking

The importance of measuring elicited emotions and their associations with product acceptability, consumption intent (CI), and PI has been emphasized because they provide information beyond liking about consumers’ eating behaviors [77,78,81,89].

**Figure 7:**
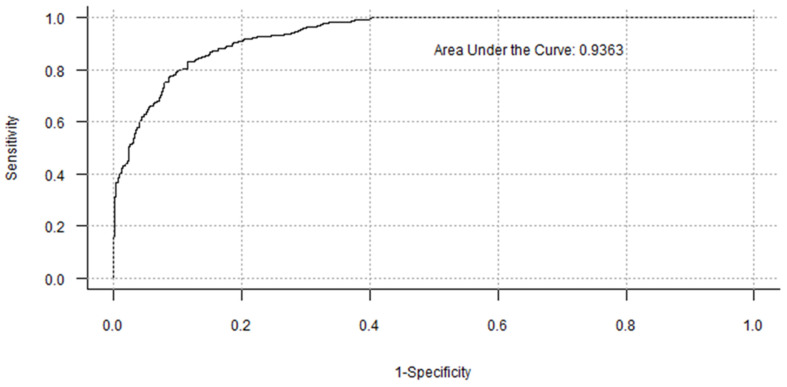
Receiver Operating Characteristic (ROC) curve illustrating the area under the curve (AUC) for the random forest classifier.

**Figure 8:**
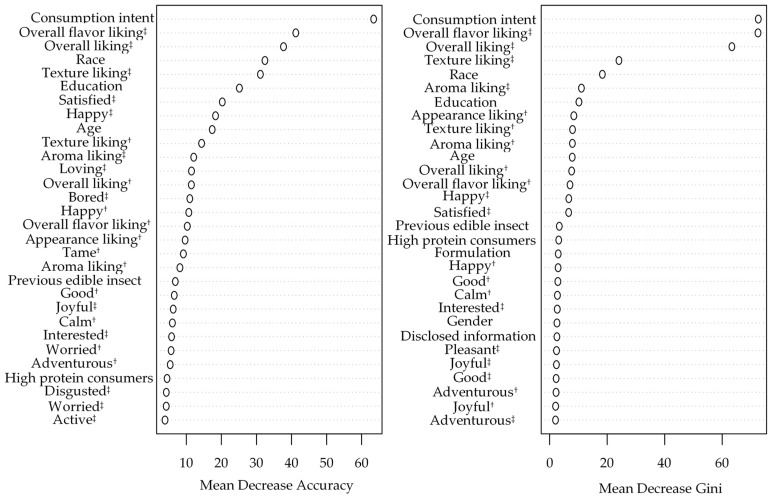
Random forest classifier variables importance plots for purchase intent (PI) prediction. ^†^ Before-tasting condition; ^‡^ after-tasting condition.

The performance of a random-forest PI prediction model using demographic variables (Table 1), likings, emotions, and experimental design variables as input is presented in Figure 7. The variables’ importance derived from this model with an out-of-bag misclassification error rate of 14.64% is presented in Figure 8. Consumption intent [50], overall flavor liking, overall liking, texture liking [90], race [89], education level [91], and expected texture liking were among the top 10 most important variables for the correct prediction of PI as determined by mean decrease in classification accuracy and mean decrease in node impurity when the variable is permuted and split, respectively. “Satisfied” and “happy” after-tasting positive-valence emotions [33,80,84,85] and age [46,51] were critical for accurate PI prediction whereas expected and actual aroma liking, and appearance liking were critical PI predictors to obtain higher node purity. Although previous edible insect consumption [50,90], formulation [8,65], gender [8,50,52,61,79], disclosed information [8,28,57,83], after-tasting disgust [22,59] and worried were considered important for the PI prediction, the aforementioned variables were more critical to determine consumers’ PI. Based on this model, the probability of purchase is higher for the consumer who is willing to consume the product upon tasting (CI = Yes), is Latino, has achieved or is pursuing a higher education degree, is satisfied and happy upon tasting, and is aged 18–29 years old. Additionally, the higher his/her liking ratings for actual overall flavor, OL, texture, aroma, appearance, and expected texture and aroma liking, the more likely the consumer purchases the product. These results suggest that marketing strategies should target consumers who match this ideal “profile,” as they are more likely to purchase CB containing ECP. Furthermore, these results highlight the importance of sensory profile optimization for products containing ECP and appropriate benefits communication that evoke positive valence emotions known to improve overall acceptability and PI.

## 4. Study Limitations

The screening and recruitment of participants for this study did not follow a pre-specified demographic criterion except for the minimum required age to participate in studies involving human subjects according to the guidelines of the Institutional Review Board of Louisiana State University Agricultural Center (IRB # HE 18-9 and IRB # HE 18-22). Hence, the participants’ age and race distributions (Table 1) neither are equally balanced nor reflect the actual distribution of the US population; therefore, the findings from this study should be interpreted with caution and should not be generalized for the entire population. Likewise, the sample size in this study (*n* = 210) was not large enough to represent the entire US population; thus, a study with a much larger sample size is needed to confirm our findings. Lastly, because this study used only one product (chocolate brownies) and one concentration (6% *w/w*) of a commercial cricket protein powder, different results can be expected if different test samples and other concentrations or sources of cricket protein powder are to be used.

## 5. Conclusions and Future Studies

A better understanding of consumers’ attitudes toward ECP and recommended approaches for incorporating edible insects into foods were achieved in this study. Actual OL was more affected by formulation than by disclosed information among the female group (showing higher acceptability for CBWO than for CBW) whereas the male group’s actual OL was similar across all treatments. Yet, the female group presented significant negative disconfirmation upon tasting only for CBW−. Disclosed information had a greater effect than formulation on product-evoked emotions (before and after tasting) with “happy,” “satisfied,” “good,” “pleasant,” and “interested” being significant drivers for actual OL in both genders whereas “mild” inhibited actual OL among the female group. Consumption intent, race, education level, positive-valence after-tasting emotions, age, and sensory liking profile were top determinants for PI prediction. Because entomophagy is a new concept for the Western culture, information regarding the consumption of edible insects, including safety (potential biological, chemical, and physical hazards), environmental impact, and nutritional benefits may improve familiarity and alleviate aversion to entomophagy. Thus, our findings may guide future development of products incorporated with ECP for the Westerner diets. From our findings, we suggest that marketing strategies for ECP bakery applications target younger Latinos with higher education as they are more likely to purchase products containing ECP. According to our results, ECP acceptability can be improved through an appropriate food application and context for ECP whose formulation is optimized for sensory liking and emphasizing benefits from ECP consumption, which in turn evokes positive-valence emotions such as “happy” and “satisfied” that positively affect OL and PI. This relationship is important to the food industry to guide them in the development and marketing of foods containing edible insects, particularly for baked goods containing ECP. Product-elicited emotions (whose distribution in the before-tasting condition was independent of gender for CBW+ but associated with gender in the after-tasting condition for CBW+) add predictive power to solely liking ratings to understand consumers’ PI behavior. This may guide the food industry in the development of “unique” products different from the ones existing in the market but with similar liking.

In the future, we recommend a consumer-based descriptive analysis to correlate the observed results with sensory descriptors and obtain additional insight as to what other sensory attribute may also affect product liking, consumers’ emotions, and PI.

## Figures and Tables

**Table 1:** Demographic profile of participants from the consumer study.

Demographic Variables	Levels	*n*	%
Gender	Female	112	53.33
	Male	98	46.67
Age group	18–22	93	44.29
	23–29	84	40.00
	30–39	24	11.43
	40–49	5	2.38
	50–59	3	1.43
	≥60	1	0.48
Race	Asian	37	17.62
	Black/African American	27	12.86
	Latino	41	19.52
	White/Caucasian	100	47.62
	Other	5	2.38
Highest education level achieved	College degree	56	26.67
	Graduate or professional degree	74	35.24
	High school or lower degree	80	38.10
High-protein products consumption	Yes	123	58.57
	No	87	41.43
Previously tasted products with edible insects	Yes	117	55.71
	No	93	44.29

**Table 2:** ANOVA ^†^ table for the overall sensory acceptability of CB treatments ^‡^.

Effects	Overall Liking ^§^	
	F Value	Pr > F
Gender	2.54	0.11
Age	1.43	0.22
Race	1.53	0.20
Education	1.32	0.27
High protein consumption	0.40	0.53
Previous edible insect	2.54	0.11
Tasting condition	17.95	<0.01
Formulation	35.00	<0.01
Disclosed information	3.07	0.08
Tasting condition * Formulation	15.58	<0.01
Tasting condition * Disclosed information	29.10	<0.01
Formulation * Disclosed information	2.84	0.09
Gender * Formulation	6.32	0.01
Gender * Disclosed information	0.35	0.55
Tasting condition * Formulation * Disclosed information	0.00	0.98
Gender * Formulation * Disclosed information	0.16	0.69

^†^ ANOVA = Analysis of variance [2 genders (female and male), 6 age groups (18–22, 23–29, 30–39, 40–49, 50–59, ≥60 years old), 5 races (Asian, Black/African American, Latino, White/Caucasian, Other), 3 education levels (college, graduate/professional degree, high school or lower degree), 2 levels of high protein consumption (yes and no), 2 levels of previous edible insect (yes and no), 2 levels of tasting condition (before and after), 2 levels of formulation (CBWO and CBW), 2 levels of disclosed information (ECP− and ECP+). ^‡^ Treatments are described in Figure 1. ^§^ Overall liking data from *n* = 210 consumers were collected using a 9-point hedonic scale (1 = dislike extremely, 9 = like extremely) and analyzed by a mixed-effects model with panelists as a random effect. * Denotes interaction.

**Table 3:** Emotional profile ^†^ elicited by treatments ^‡^ in the before-tasting condition.

Emotions	Females				Males			
	CBWO−	CBWO+	CBW−	CBW+	CBWO−	CBWO+	CBW−	CBW+
Active	13 ^A^	9 ^A^	10 ^A^	8 ^A^	10 ^a^	15 ^a^	7 ^a^	11 ^a^
Adventurous	11 ^B^	***47 ^A^***	10 ^B^	***43 ^A^***	7 ^b^	30 ^a^	11 ^b^	***39 ^a^***
Aggressive	3 ^A^	4 ^A^	4 ^A^	4 ^A^	2 ^a^	1 ^a^	1 ^a^	2 ^a^
Bored	8 ^B^	1 ^B^	20 ^A^	5 ^B^	8 ^a,b^	4 ^a,b^	11 ^a^	3 ^b^
Calm	31 ^A^	15 ^B^	25 ^A,B^	14 ^B^	27 ^a^	19 ^a,b^	28 ^a^	15 ^b^
Disgusted	0 ^B^	8 ^A^	1 ^A,B^	8 ^A^	2 ^a^	5 ^a^	4 ^a^	5 ^a^
Enthusiastic	***20 ^A^***	***29 ^A^***	***18 ^A^***	***25 ^A^***	14 ^a^	19 ^a^	16 ^a^	21 ^a^
Free	11 ^A^	7 ^A^	7 ^A^	***8 ^A^***	8 ^a^	13 ^a^	8 ^a^	10 ^a^
Good	47 ^A,B^	40 ^A,B^	***51 ^A^***	34 ^B^	50 ^a^	43 ^a,b^	41 ^a,b^	35 ^b^
Good natured	10 ^A^	12 ^A^	8 ^A^	11 ^A^	10 ^a^	16 ^a^	13 ^a^	12 ^a^
Guilty	4 ^A^	4 ^A^	5 ^A^	6 ^A^	5 ^a^	3 ^a^	5 ^a^	3 ^a^
Happy	32 ^A,B^	20 ^B,C^	34 ^A^	13 ^C^	27 ^a^	17 ^a^	20 ^a^	24 ^a^
Interested	***46 ^B^***	***71 ^A^***	***39 ^B^***	***74 ^A^***	***45 ^a^***	***53 ^a^***	***40 ^a^***	***51 ^a^***
Joyful	21 ^A^	13 ^A,B^	13 ^A,B^	9 ^B^	16 ^a^	12 ^a^	12 ^a^	8 ^a^
Loving	6 ^A^	4 ^A^	6 ^A^	6 ^A^	7 ^a^	4 ^a^	6 ^a^	4 ^a^
Mild	28 ^A^	23 ^A^	26 ^A^	23 ^A^	11 ^a^	12 ^a^	20 ^a^	12 ^a^
Nostalgic	7 ^A,B^	4 ^B^	11 ^A^	4 ^B^	6 ^a^	4 ^a^	5 ^a^	2 ^a^
Pleasant	27 ^A^	13 ^B^	***31 ^A^***	8 ^B^	21 ^a^	15 ^a^	27 ^a^	20 ^a^
Safe	29 ^A,B^	16 ^B,C^	***33 ^A^***	11 ^C^	18 ^a,b^	17 ^a,b^	23 ^a^	9 ^b^
Satisfied	23 ^A^	11 ^A,B^	21 ^A,B^	10 ^B^	15 ^a^	10 ^a^	13 ^a^	10 ^a^
Tame	11 ^A^	2 ^B^	9 ^A,B^	5 ^A,B^	6 ^a^	5 ^a^	7 ^a^	4 ^a^
Understanding	4 ^A^	***13 ^A^***	5 ^A^	10 ^A^	4 ^a^	9 ^a^	6 ^a^	9 ^a^
Warm	***17 ^A^***	6 ^B^	11 ^A,B^	5 ^B^	***14 ^a^***	4 ^b^	11 ^a,b^	6 ^a,b^
Wild	2 ^B^	***12 ^A^***	3 ^B^	***12 ^A^***	2 ^c^	11 ^a,b^	3 ^b,c^	13 ^a^
Worried	3 ^C^	***13 ^A,B^***	5 ^B,C^	***15 ^A^***	4 ^a,b^	5 ^a,b^	1 ^b^	8 ^a^

^†^ Frequency of emotions in the before-tasting condition from *n* = 112 female and *n* = 98 male groups analyzed by two-sided Cochran’s Q test with Marascuilo and McSweeney procedure (multiple-pairwise-comparisons-minimum-required difference) [44]. Different uppercase/lowercase letters within a row represent significant (*p* < 0.05) differences in the female/male group’s emotion across treatments. Bolded and italicized frequency was significantly (*p* < 0.05) higher than its corresponding emotion in the after-tasting condition (Table 4). ^‡^ Treatments are described in Figure 1.

**Table 4:** Emotional profile ^†^ elicited by treatments ^‡^ in the after-tasting condition.

Emotions	Females				Males			
	CBWO−	CBWO+	CBW−	CBW+	CBWO−	CBWO+	CBW−	CBW+
Active	9 ^A^	5 ^A^	9 ^A^	5 ^A^	6 ^a^	10 ^a^	6 ^a^	8 ^a^
Adventurous	7 ^B^	35 ^A^	9 ^B^	28 ^A^	6 ^b^	25 ^a^	8 ^b^	23 ^a^
Aggressive	2 ^A^	4 ^A^	2 ^A^	4 ^A^	1 ^a^	1 ^a^	0 ^a^	2 ^a^
Bored	***18 ^A^***	***6 ^B^***	17 ^A,B^	11 ^A,B^	11 ^a,b^	7 ^a,b^	15 ^a^	6 ^b^
Calm	25 ^A^	12 ^B^	18 ^A,B^	18 ^A,B^	30 ^a^	22 ^a,b^	20 ^a,b^	18 ^b^
Disgusted	***4 ^A^***	9 ^A^	***13 ^A^***	14 ^A^	3 ^a^	4 ^a^	7 ^a^	7 ^a^
Enthusiastic	8 ^A^	10 ^A^	10 ^A^	8 ^A^	8 ^a^	12 ^a^	10 ^a^	14 ^a^
Free	6 ^A,B^	4 ^A,B^	8 ^A^	1 ^B^	10 ^a^	11 ^a^	8 ^a^	10 ^a^
Good	53 ^A,B^	***56 ^A^***	39 ^B,C^	36 ^C^	44 ^a^	42 ^a^	44 ^a^	***50 ^a^***
Good natured	6 ^A^	11 ^A^	9 ^A^	6 ^A^	15 ^a^	20 ^a^	10 ^a^	16 ^a^
Guilty	5 ^A^	4 ^A^	4 ^A^	5 ^A^	3 ^a^	3 ^a^	3 ^a^	3 ^a^
Happy	38 ^A^	26 ^A,B^	29 ^A,B^	17 ^B^	35 ^a^	23 ^a^	27 ^a^	26 ^a^
Interested	28 ^B^	48 ^A^	25 ^B^	49 ^A^	21 ^a^	33 ^a^	24 ^a^	28 ^a^
Joyful	19 ^A^	18 ^A^	13 ^A^	9 ^A^	14 ^a^	15 ^a^	16 ^a^	***16 ^a^***
Loving	6 ^A^	8 ^A^	8 ^A^	3 ^A^	6 ^a^	5 ^a^	8 ^a^	5 ^a^
Mild	23 ^A^	28 ^A^	30 ^A^	23 ^A^	***23 ^a^***	14 ^a^	20 ^a^	17 ^a^
Nostalgic	13 ^A^	***10 ^A,B^***	8 ^A,B^	4 ^B^	8 ^a^	5 ^a^	6 ^a^	4 ^a^
Pleasant	31 ^A^	***29 ^A^***	21 ^A^	***24 ^A^***	31 ^a^	***39 ^a^***	30 ^a^	26 ^a^
Safe	25 ^A^	17 ^A^	13 ^A^	17 ^A^	15 ^a^	18 ^a^	18 ^a^	15 ^a^
Satisfied	***37 ^A^***	***39 ^A^***	26 ^A^	***39 ^A^***	***36 ^a^***	***41 ^a^***	***31 ^a^***	***30 ^a^***
Tame	11 ^A^	5 ^A^	11 ^A^	6 ^A^	10 ^a^	7 ^a^	***14 ^a^***	9 ^a^
Understanding	4 ^A,B^	5 ^A,B^	1 ^B^	10 ^A^	5 ^a^	9 ^a^	5 ^a^	10 ^a^
Warm	9 ^A^	6 ^A^	7 ^A^	9 ^A^	8 ^a^	8 ^a^	10 ^a^	6 ^a^
Wild	4 ^A^	5 ^A^	4 ^A^	2 ^A^	2 ^b^	11 ^a^	2 ^b^	11 ^a^
Worried	1 ^B^	6 ^A,B^	7 ^A,B^	8 ^A^	1 ^a^	3 ^a^	2 ^a^	3 ^a^

^†^ Frequency of emotions in the after-tasting condition from *n* = 112 female and *n* = 98 male groups analyzed by two-sided Cochran’s Q test with Marascuilo and McSweeney procedure (multiple-pairwise-comparisons-minimum-required difference) [44]. Different uppercase/lowercase letters within a row represent significant (*p* < 0.05) differences in the female/male group’s emotion across treatments. Bolded and italicized frequency was significantly (*p* < 0.05) higher than its corresponding emotion in the before-tasting condition (Table 3). ^‡^ Treatments are described in Figure 1.

## Data Availability

The data that support the findings of this study are available from the corresponding author upon reasonable request.
